# Supplementary material for: Structural basis for the regulation of plant transcription factor WRKY33 by the VQ protein SIB1
Source: Commun Biol. 2024 May 11;7:561. doi: 10.1038/s42003-024-06258-7 (PMC11088704; doi:10.1038/s42003-024-06258-7)

Supplemental Information for

"Structural basis for the regulation of plant transcription factor WRKY33 by the VQ protein SIB1"

This file includes Supplemental Figures S1-S16.

Supplemental Figures:

a

```
         10         20         30         40         50
MESSSSTFLT TTSLDKKKPS PVSRKSPKQK KKTTSTNKPI KVRYISNPMR
         60         70         80         90        100
VQTCASKFRE LVQELTGQDA VDLQPEPIYS PSSDDHNLSP PAENLAPRVL
        110        120        130        140        150
HQEPFGERDS DCYEPLNAED MFLPDQMSAG FSGFFSNGFY NVNDFGSIDS

M
```

Chloroplast localization signal peptide (SP)

Nuclear locallization signal (NLS)

C-terminal region (C-ter)

b

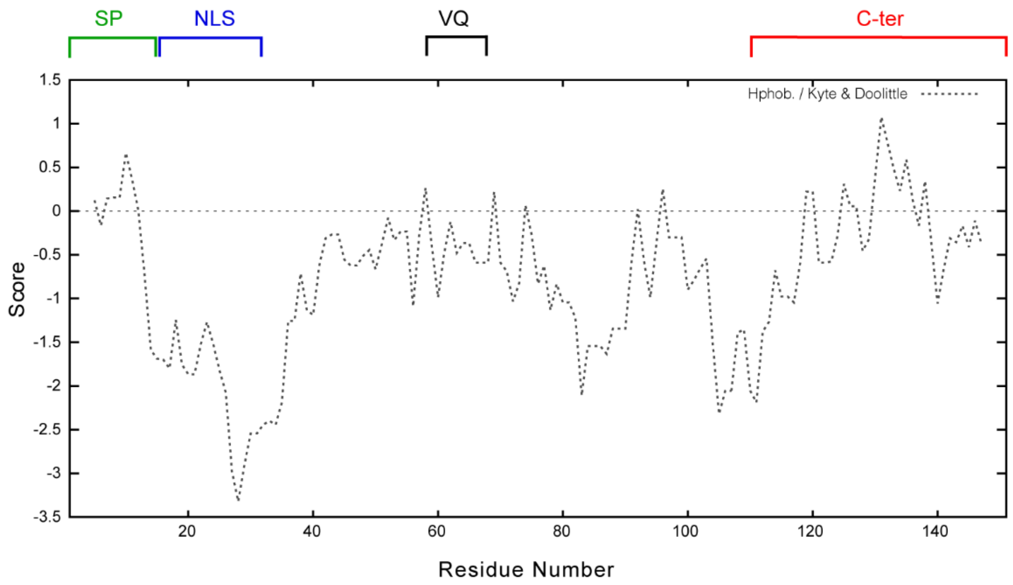

Figure S1. Primary sequence and hydrophobicity analysis of SIB1. (a) Primary sequence of full-length SIB1 showing the predicted chloroplast targeting signal peptide (green), the nuclear localization signal (blue) and the C-terminal 51 residues truncated in our study (red). **(b)** Hydrophobicity analysis of full-length SIB1 using the ProtScale tool on the ExPASy server (https://web.expasy.org/protscale/).

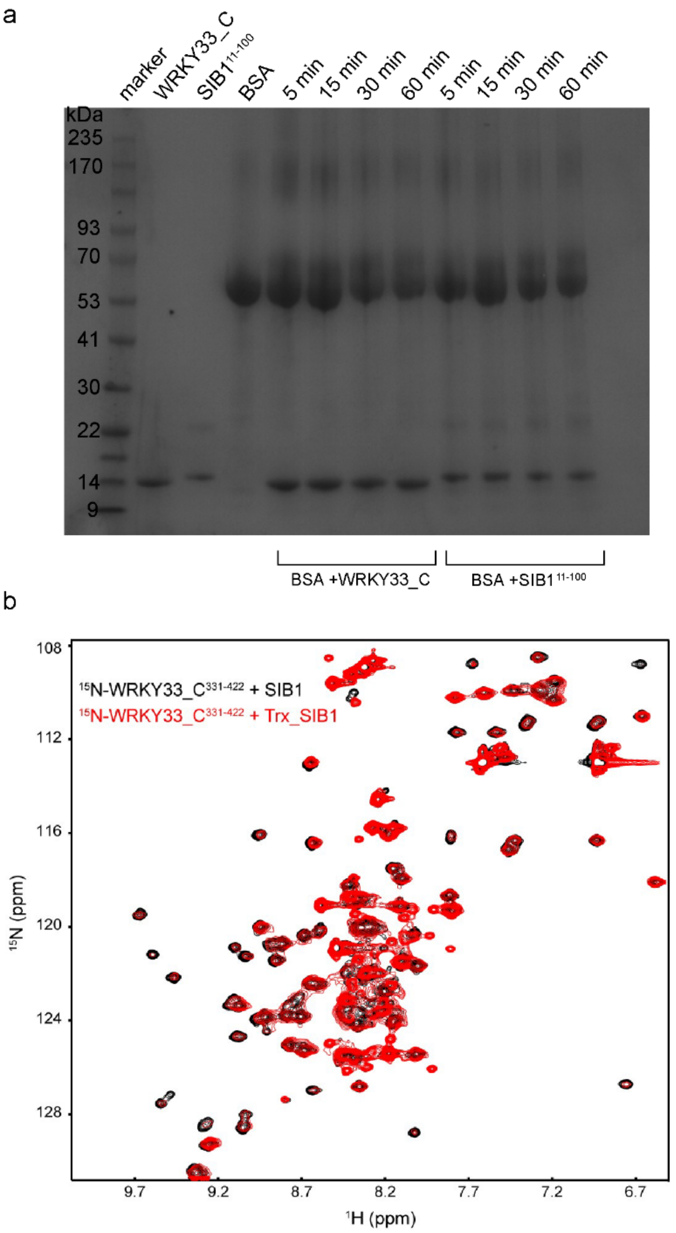

Figure S2. ***In vitro*** **interaction between SIB1 and WRKY33_C.** (**a**) Control experiment showing the EGS cross-linking results between WRKY33_C/SIB1$^{11\text{-}100}$ and BSA. (**b**) An overlay of the 2D ^{1}H–^{15}N HSQC spectra of ^{15}N-labeled WRKY33_C in complex with unlabeled SIB1 (black) or Trx_SIB1 (red), showing that Trx_SIB1 binds WRKY33_C the same way as SIB1.

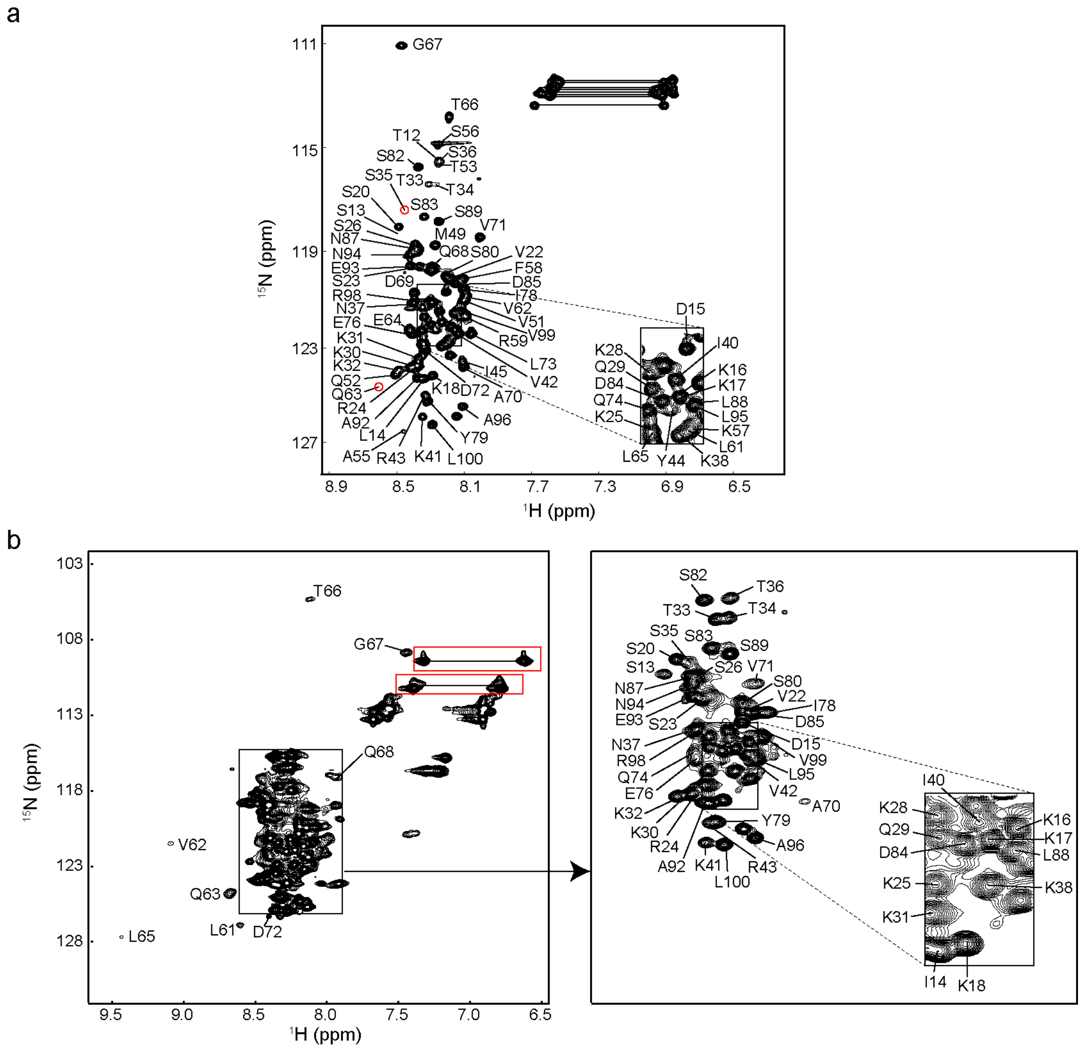

Figure S3. Backbone assignments of SIB1 in the free and the complexed states. (**a**) ^{1}H-^{15}N HSQC spectrum of ^{15}N-labeled SIB1 in the free state annotated with backbone assignments. The small rectangular box shows enlarged view of the central region. Pairs of side chain NH_2 peaks of Asn and Gln residues are connected by lines. The red empty circles indicate the presence of weak signals that are not observable at the current display level. (**b**) The ^{1}H–^{15}N HSQC spectra of ^{15}N-labeled SIB1 in the WRKY33_C-complexed state annotated with backbone assignments. The pairs of side chain NH_2 peaks of Asn and Gln residues are connected by lines. The red rectangular boxes indicate the two pairs of newly appeared NH_2 signals, which were identified to correspond to side chains of Gln63 and Gln68 from the VQ motif based on mutagenesis.

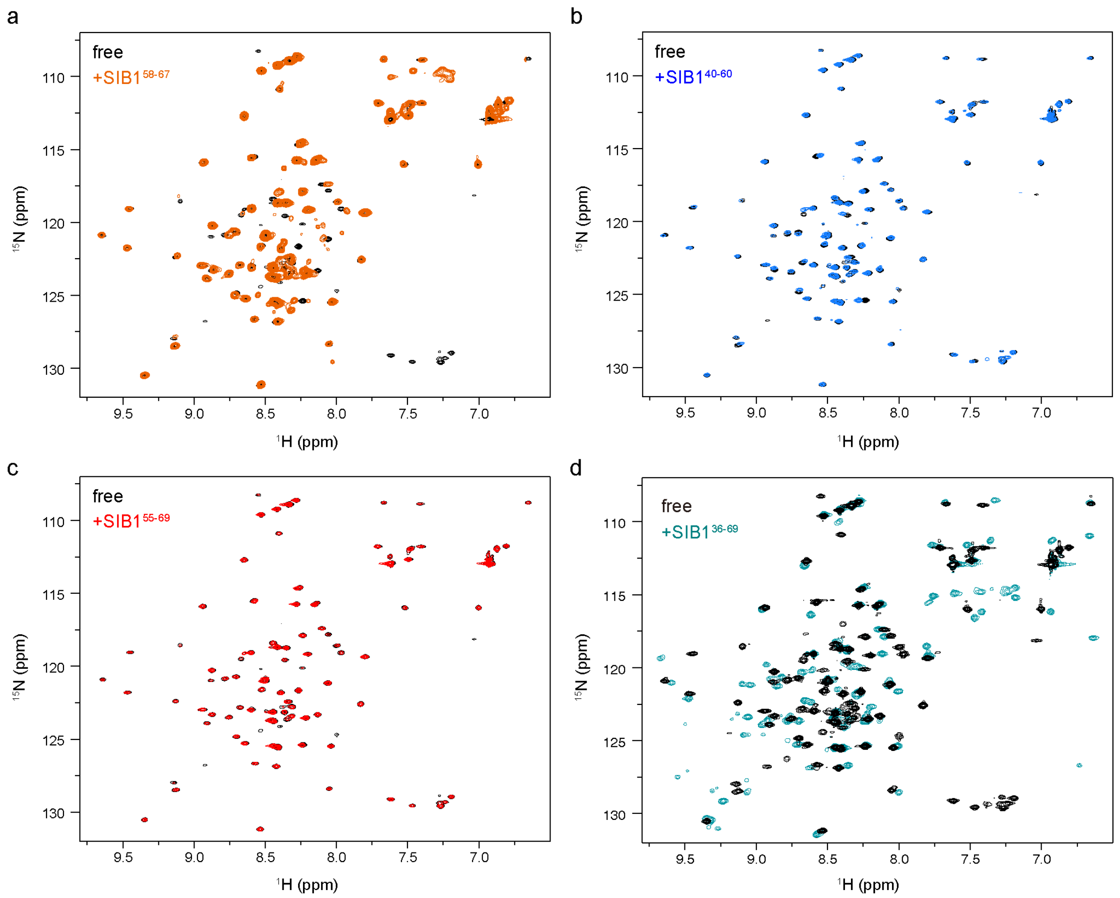

Figure S4. NMR-monitored interaction between ^{15}N-WRKY33_C and different SIB1 segments. Overlay of the HSQC spectra of ^{15}N-labeled WRKY33_C (100 μM) in its free state (black) and in the presence of 2-fold excess of SIB1$^{58\text{-}67}$(a), SIB1$^{40\text{-}60}$ (b), SIB1$^{55\text{-}69}$ (c) or SIB1$^{36\text{-}69}$ (d).

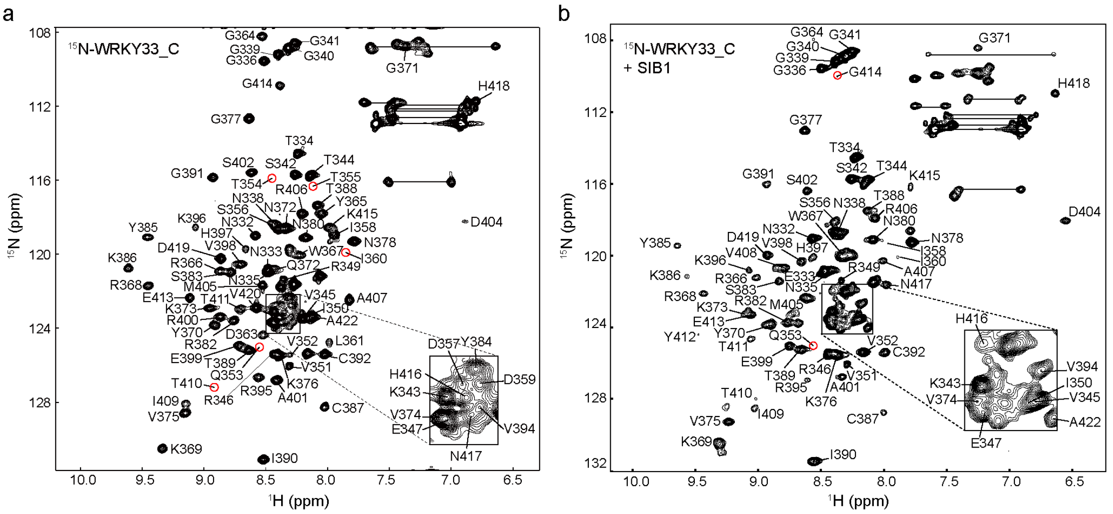

Figure S5. Backbone chemical shift assignments of WRKY33_C in the free and SIB1-complexed states. 2D ^{1}H–^{15}N HSQC spectra of ^{15}N-labeled WRKY33_C in the free (a) and SIB1-complexed (b) states are annotated with backbone assignments. The pairs of side chain NH_2 peaks of Asn and Gln residues are connected by lines. The red empty circles indicate the presence of weak signals that are not observable at the current display level.

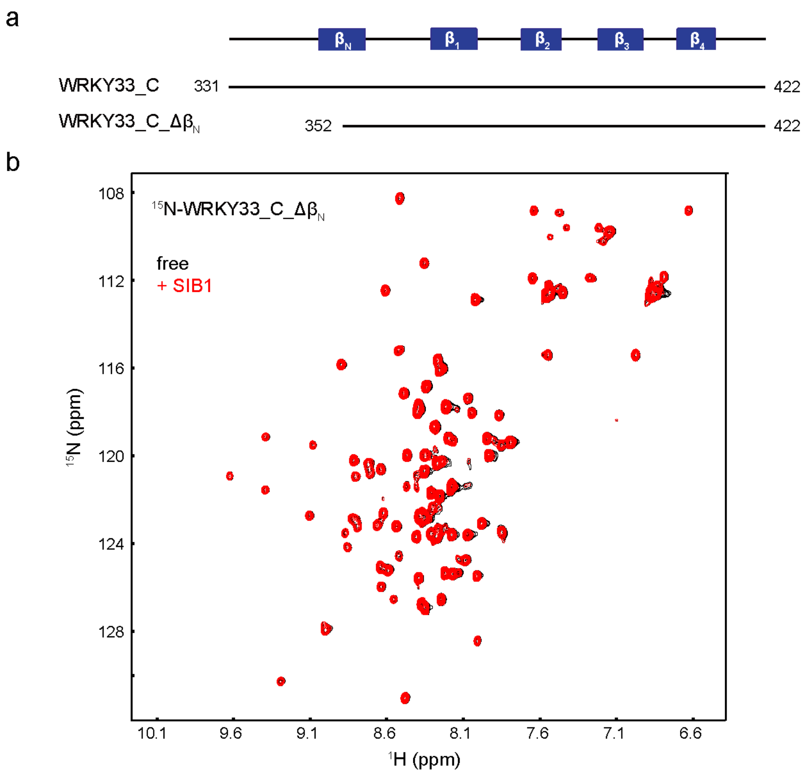

Figure S6. Characterization of the WRKY33_C_Δβ$_N$ construct. (**a**) Illustration of the construction of the WRKY33_C_Δβ$_N$ construct. (**b**) Overlay of the ^{1}H-^{15}N HSQC spectra of the ^{15}N-WRKY33_C_Δβ$_N$ in its free state (*black*) or in the presence of excess unlabeled SIB1 (*red*).

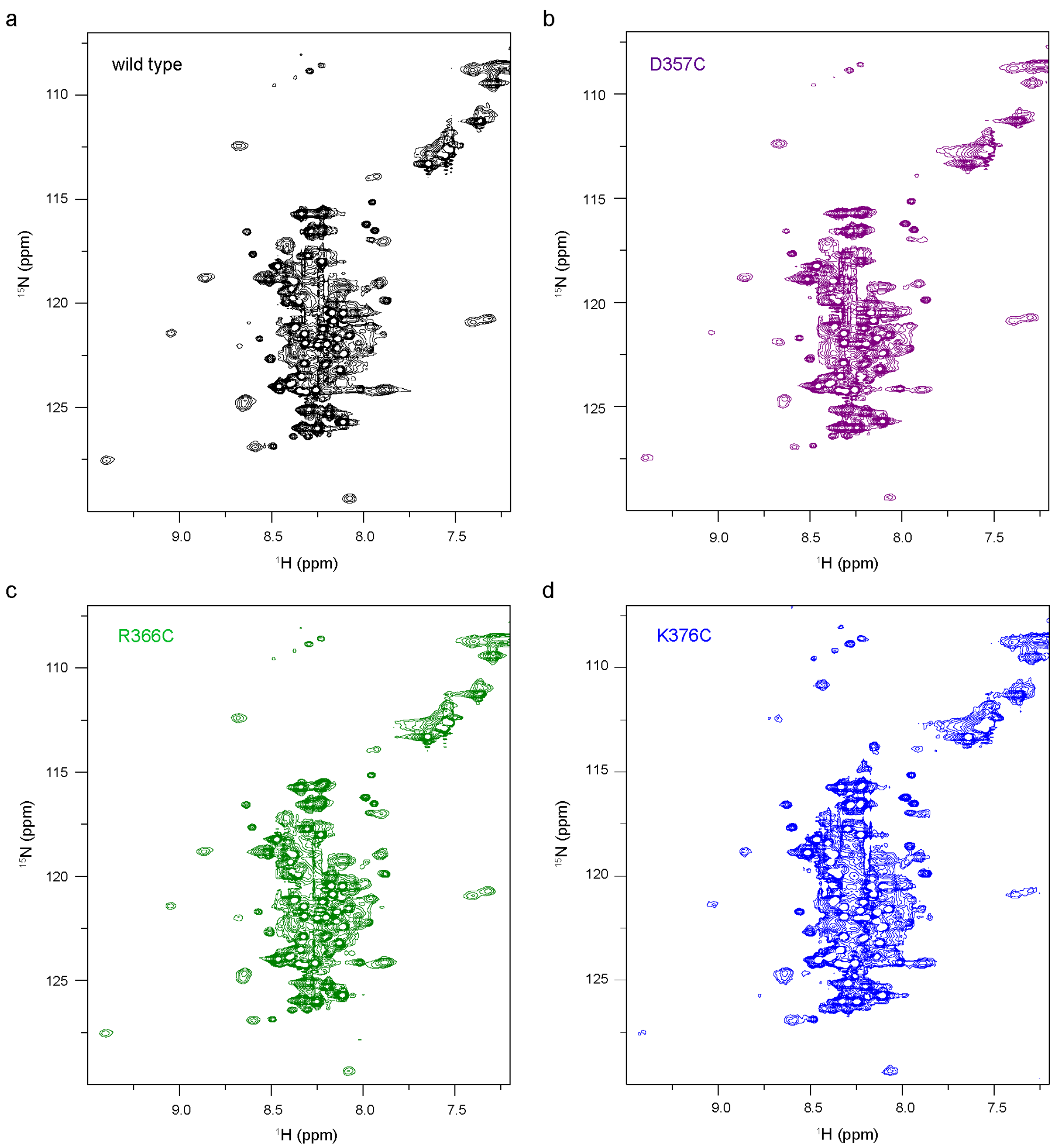

Figure S7. Spectral comparison of ^{15}N-labeled SIB1 in complex with wild-type WRKY33_C or the spin-labeled mutants. The ^{1}H-^{15}N HSQC spectra of ^{15}N-SIB1 in the presence of WRKY33_C mutants spin-labeled at the wild type (a), D357C (b), R366C (c) and K376C (d) sites.

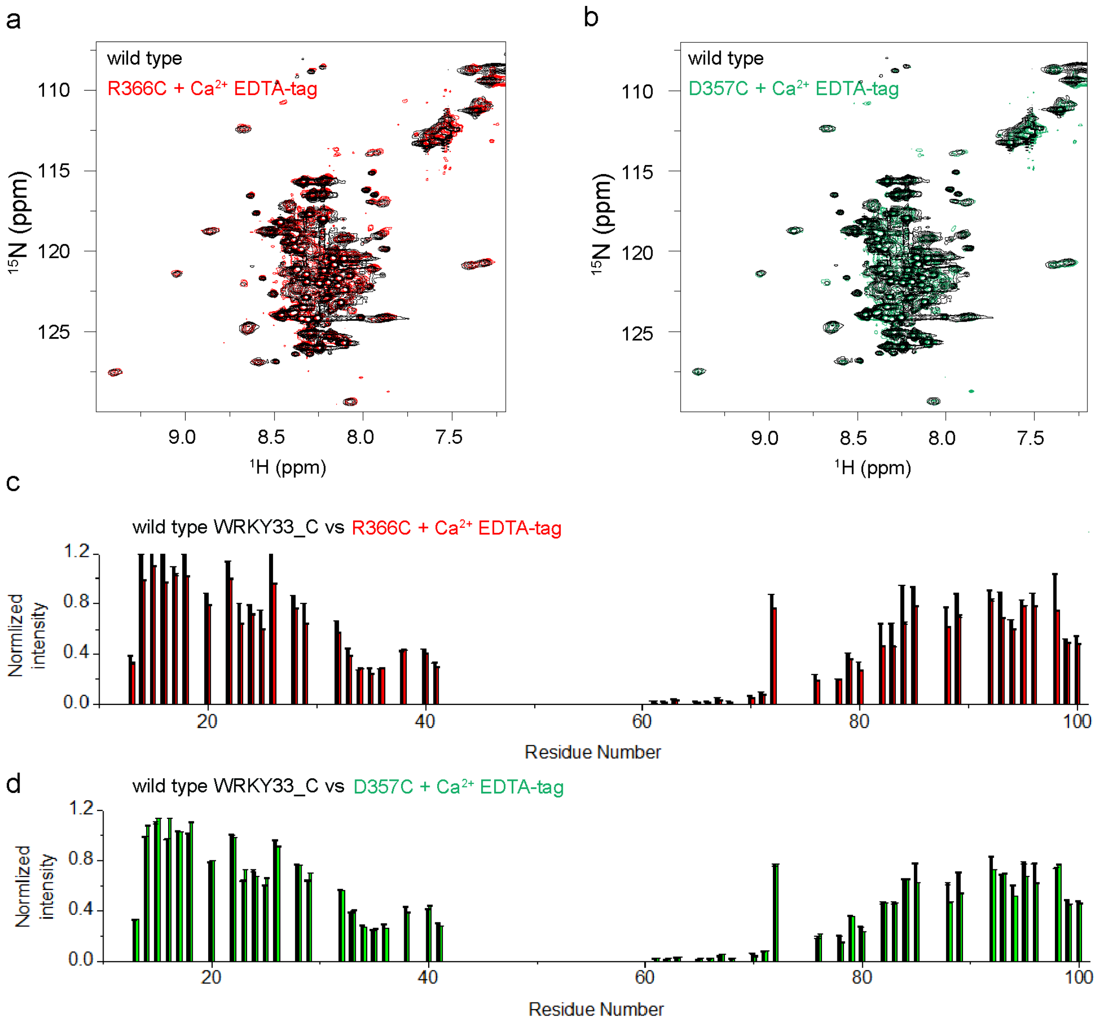

Figure S8. Comparison of the ^{1}H-^{15}N HSQC spectra of ^{15}N-labeled SIB1 in complex with wild type WRKY33_C or Ca^{2+}-EDTA tagged WRKY33_C mutants. (a-b) The overlaid ^{1}H-^{15}N HSQC spectra of ^{15}N-labeled SIB1 with wild-type WRKY33_C (black) or with Ca^{2+}-EDTA-tagged WRKY33_C-R366C mutant (red) or Ca^{2+}-EDTA-tagged WRKY33_C-D357C mutant (green). (c-d) Normalized backbone amide signal intensity profiles of the ^{15}N-labeled SIB1 samples in complex with wild-type WRKY33_C (black), or with Ca^{2+}-EDTA-tagged WRKY33_C-R366C mutant (red), or Ca^{2+}-EDTA-tagged WRKY33_C-D357C mutant (green).

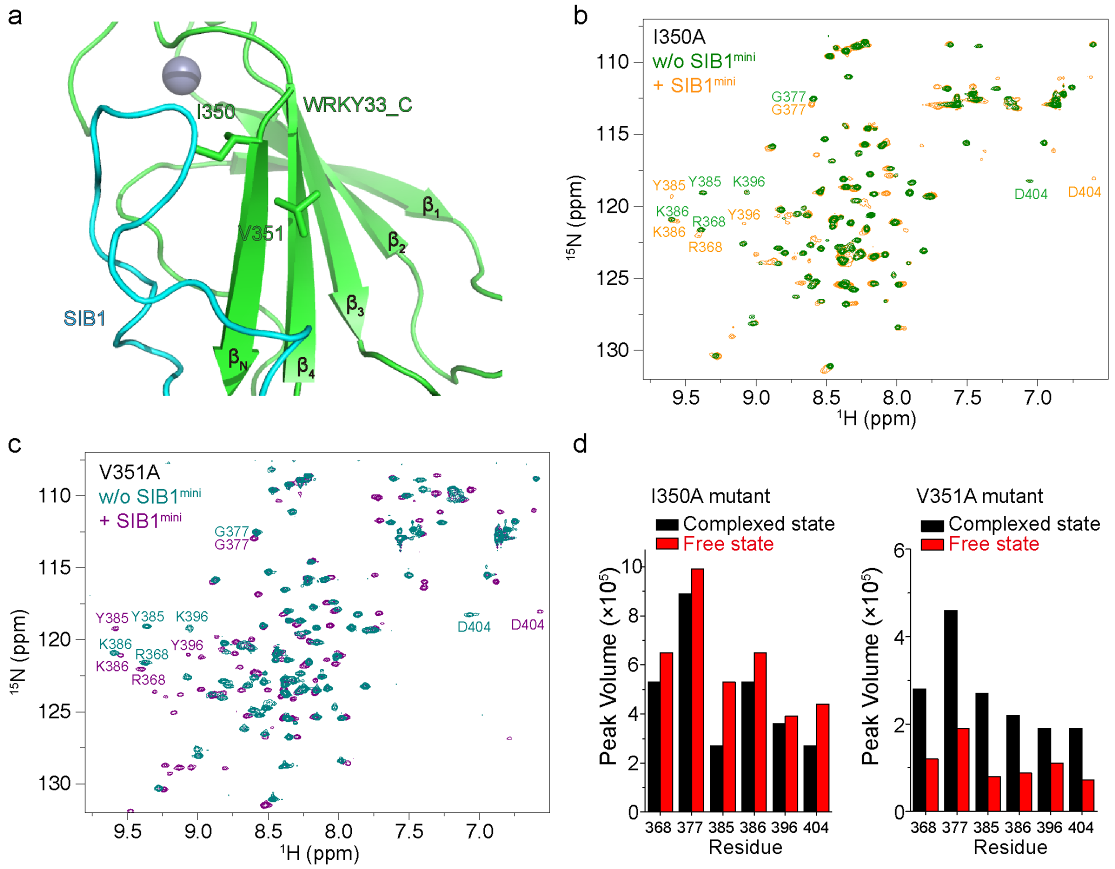

Figure S9. Mutation of hydrophobic residues in WRKY33_C β_N strand affects interaction with SIB1. (**a**) Sidechain locations of residues I350 and V351 in the β_N strand of WRKY33_C shown in a representative structure model of the SIB1-WRKY33_C complex. (**b-c**) The overlaid ^{1}H-^{15}N HSQC spectra of ^{15}N-labeled WRKY33_C I350A (**b**) or V351A (**c**) mutant in the free state and in the presence of 2-fold excess of SIB1^{mini}. Representative residues (e.g. G377, R368, Y385, K386, K396 and D404) showing two sets of resonances in the presence of SIB1^{mini} are labeled. (**d**) Peak volume analysis of the two sets of peaks of the representative resonances in the presence of SIB1^{mini}.

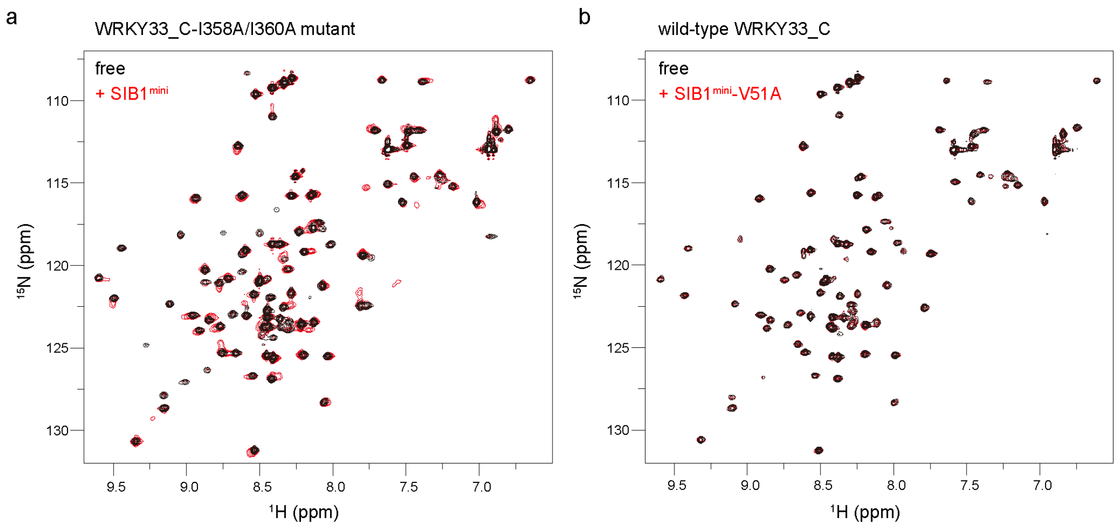

Figure S10. Residues I358/I360 in WRKY33_C and V51 in SIB1 are important for interaction. (**a**) The superimposed ^{1}H-^{15}N HSQC spectra of ^{15}N-labeled WRKY33_C-I358A/I360A double mutant in its free state (*black*) and in the presence of SIB1^{mini} (*red*). (**b**) The superimposed ^{1}H-^{15}N HSQC spectra of ^{15}N-labeled wild-type WRKY33_C in its free state (*black*) and in the presence of SIB1^{mini}-V51A mutant peptide (*red*).

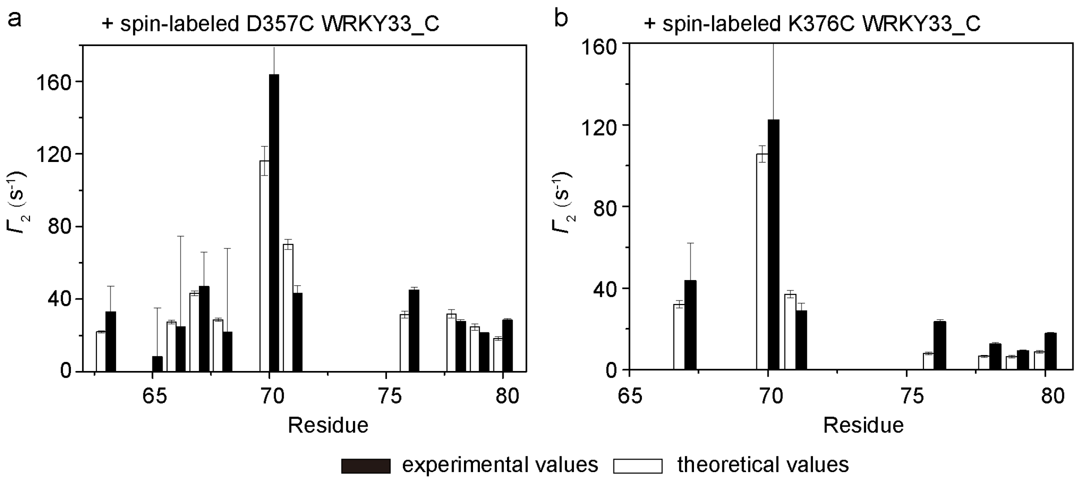

Figure S11. Comparison between the theoretical and the experimental Γ_2 rates for the SIB1-WRKY33_C complex. The experimental and the theoretical distance restraints derived from spin-labeled D357C (a) and spin-labeled K376C (b) WRKY33_C samples shown as colunms, respectively. The theoretical values are calculated as the average of the distance extracted from the 20 selected complex structures, and the error bars show the standard deviations.

```
VQ4         MENSPRYREATNLIPSPRCHNSNNSCGMSSSS--------ESNKPPTTP---------TR  43
VQ10        ---------------------------MSGRG-----------K----------------  6
MKS1        MDPSEYFAG-GNPSDQQNQKRQLQICGPRPSPLSVHKDSHKIKKPPKHPAPPPNRDQPPP  59
SIB1        MES-------------------------SSSTFLTTT-SLDKKKPSPVSRKSPKQKKKTT  34
SIB2        MDQ-------------------------SSSTLLINQ----RKSSSSPTRIPPKQKRKST  31
                                                       .

VQ4         HVTTRSESGNPYPTTFVQADTSSFKQVVQMLTGSAERPKHGSSLKPNPTHHQPDPRSTPS  103
VQ10        -VKSEPMKVVFINTQYVETDARSFKTVVQELTGKNAIVAAGPFDSP-------------S  52
MKS1        YIPREPVVIYAVSPKVVHATASEFMNVVQRLTGISSGVFLESGGG---GDVSPAARL-AS  115
SIB1        STNKPIKVRYISNPMRVQTCASKFRELVQELTGQDAVDLQPEPIY---SPSSDDHNLSPP  91
SIB2        TTHKPIKVRYISNPMRVETCPSKFRELVQELTGQDAADLPPSPTT---FTAVDLHRPCES  88
                            *.:   .*  :** ***

VQ4         SFSIPPIKAV----------PNKKQSS-SSASGF---------RLYERRNSMKNLKINPL  143
VQ10        AFDGRCYDGGSK------IGEDTRQLHGGGGGGGRMGTTTEFDRLFKEMPHMEE-----L  101
MKS1        TENASPRGGKEPAARDETVEINTAMEEAAEFGGYAPGILSPSPALLP----------TAS  165
SIB1        AENLAPRVL-----------------HQEPFGERDS------------------------  110
SIB2        EMNLEP-----------------------LDGEVRG------------------------  101
              .                            .

VQ4         NPVFNPVN---SAFSPRKPEI-LSPSI-------LDFPSLVLSPV-TPLIPDPFDRSGSS  191
VQ10        YKLWSE-------Y----------------------------------------------  108
MKS1        TGIFSPMYHQGGMFSPAIPLGLFSPAGFMSPFRSPGFTSLVASPTFADFFSHIWDQD---  222
SIB1        -DCYEPLNAEDMFLPDQMSAG------F-SGFFSNGFYNVNDFGSIDSM-----------  151
SIB2        -EYYSPLD-EEVFNAPQMSAG------L-SGFFSSGFYNVNALGSIGSL-----------  141
               :.

VQ4         NQSPNELAAEEKA
```

Figure S12. Sequence comparison of five VQ proteins that interact with WRKY33. The consensus VQ motif is indicated in red box. The positive and negative charged residues are colored blue and red in all sequences, respectively.

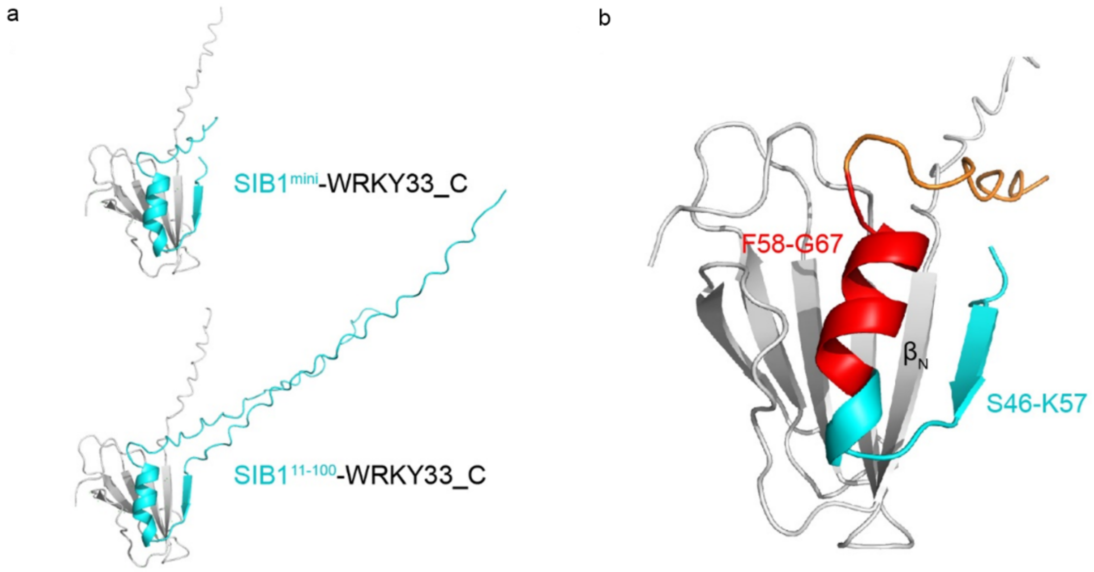

Figure S13. SIB1-WRKY33_C complex model predicted by AlphaFold2. (**a**) Cartoon representations showing the full structure of the SIB1^{mini}-WRKY33_C and SIB1$^{11\text{-}100}$-WRKY33 complex predicted using AlphaFold2. The WRKY33_C and SIB1 moleculres are shown in white and cyan, respectively. (**b**) Details of packing and binding interface between SIB1 and WRKY33_C predited by AlphaFold2. The S46-K57 and the F58-G67 segments are colored in cyan and red, respectively.

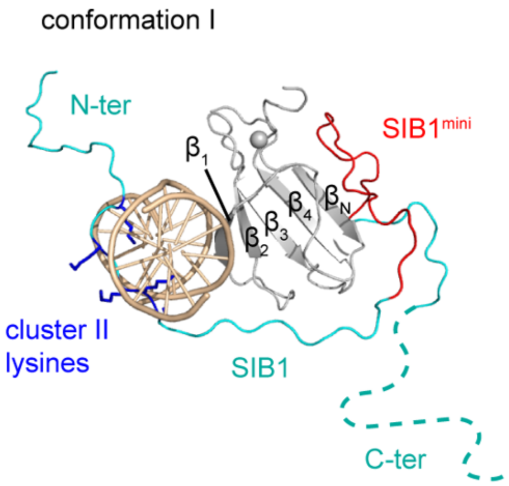

Figure S14. Cartoon representation of two major conformations of the SIB1-WRKY33_C-DNA ternary working model. The SIB1^{mini} sequence is colored in red and the remaining regions are colored in cyan. The flexible C-terminal residues (beyond 81) of SIB1 are shown as dashed lines. The side chains of cluster II lysine residues are displayed as sticks and colored in blue.

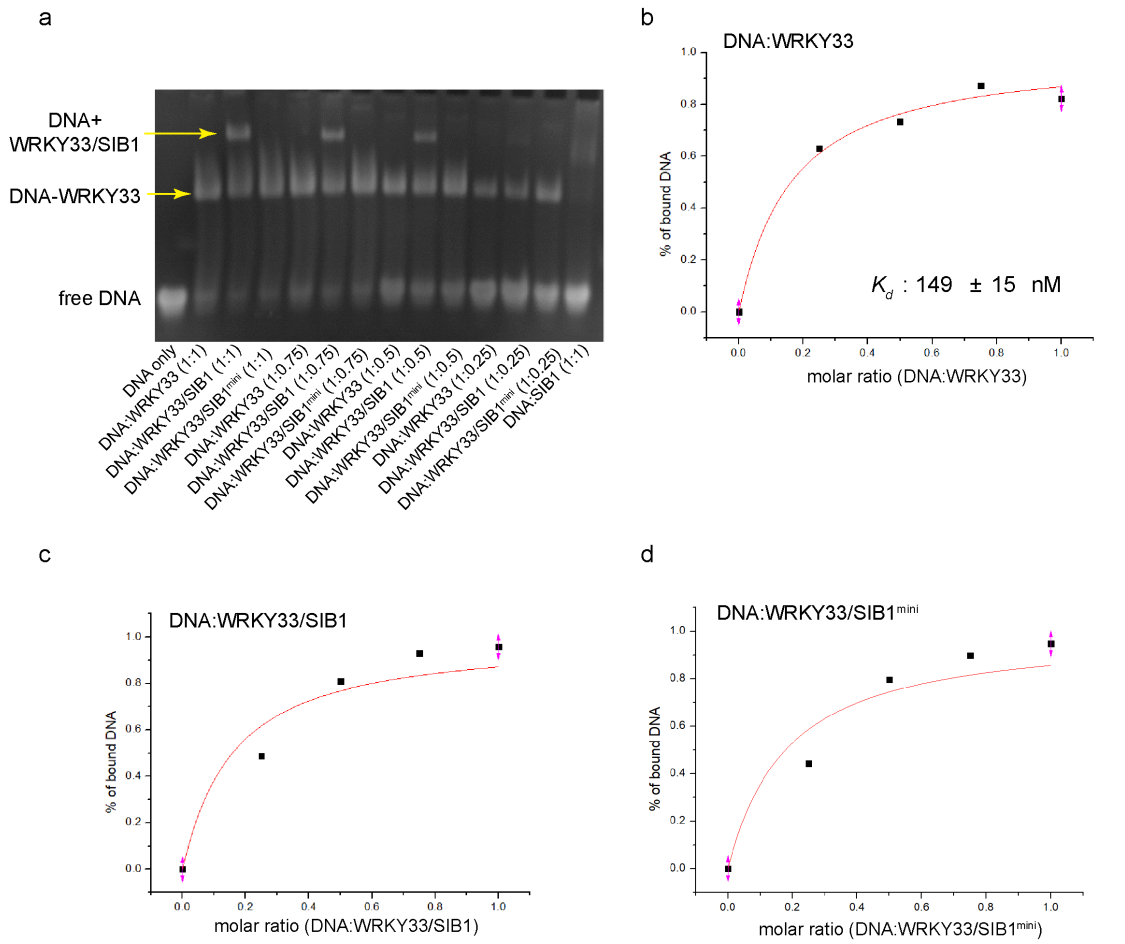

Figure S15. EMSA assays for investigating the interactions between WRKY33_C-DNA and WRKY33_C/SIB1-DNA. (**a**) The gel-image of EMSA assays of the binding between W-box DNA and WRKY33_C or WRKY33_C/SIB1 binary complex. (**b-d**) The binding curves based on band brightness. The apparent K_d value between WRKY33_C and DNA is estimated.

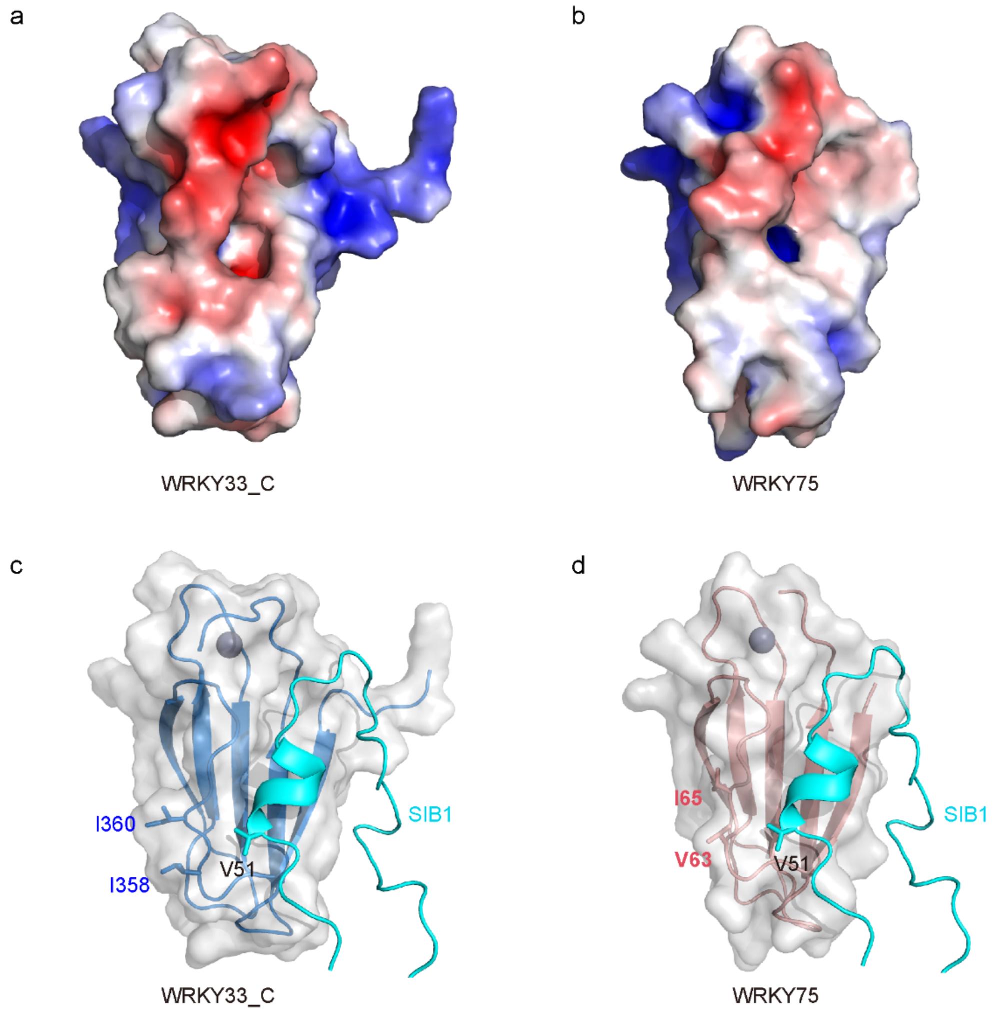

Figure S16. **Structural comparison between WRKY33_C and WRKY75**. (**a-b**) Surface electrostatic distrubutions of WRKY33_C (**a**) and WRKY75 (**b**) showing the SIB1 binding site. (**d-d**) Ribbon diagrams of the SIB1-WRKY33_C complex (**c**) and the hypothesized SIB1-WRKY75 complex (**d**). The WRKY75 structural model was built using SWISS-PROT, and the hypothezied SIB1-WRKY75 complex structural model shown in (d) was generated by aligning the WRKY75 structural model with the SIB1-WRKY33_C complex structure.

Unedited gel images:

Fig. 1b:

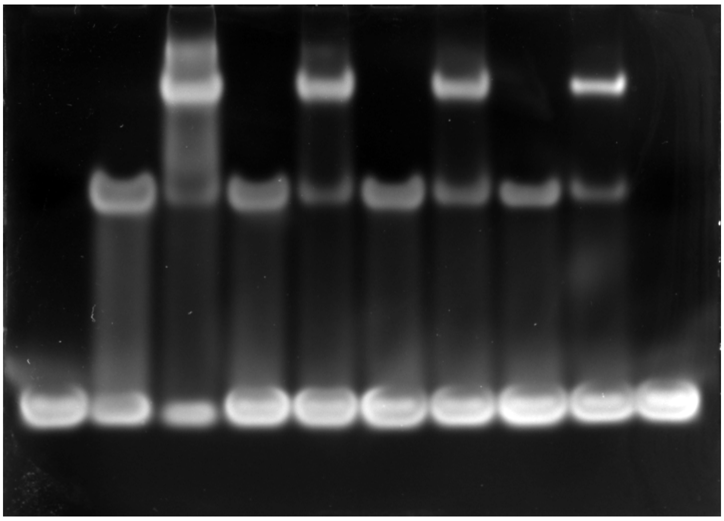

Fig. 1c :

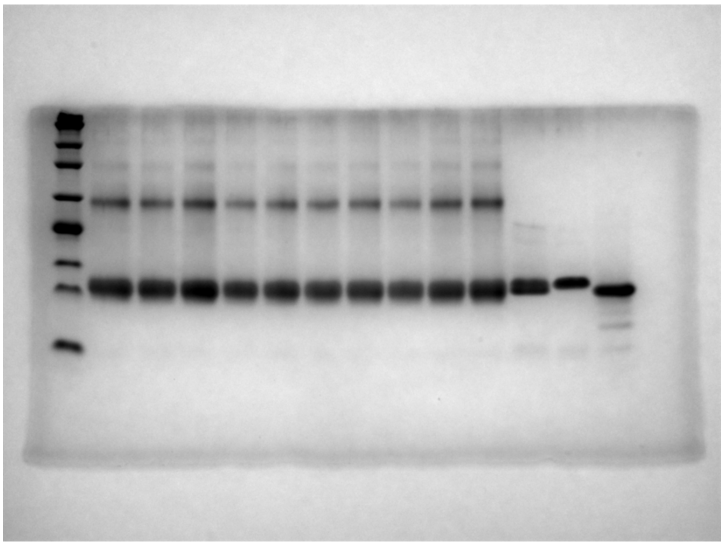

Fig. 7b:

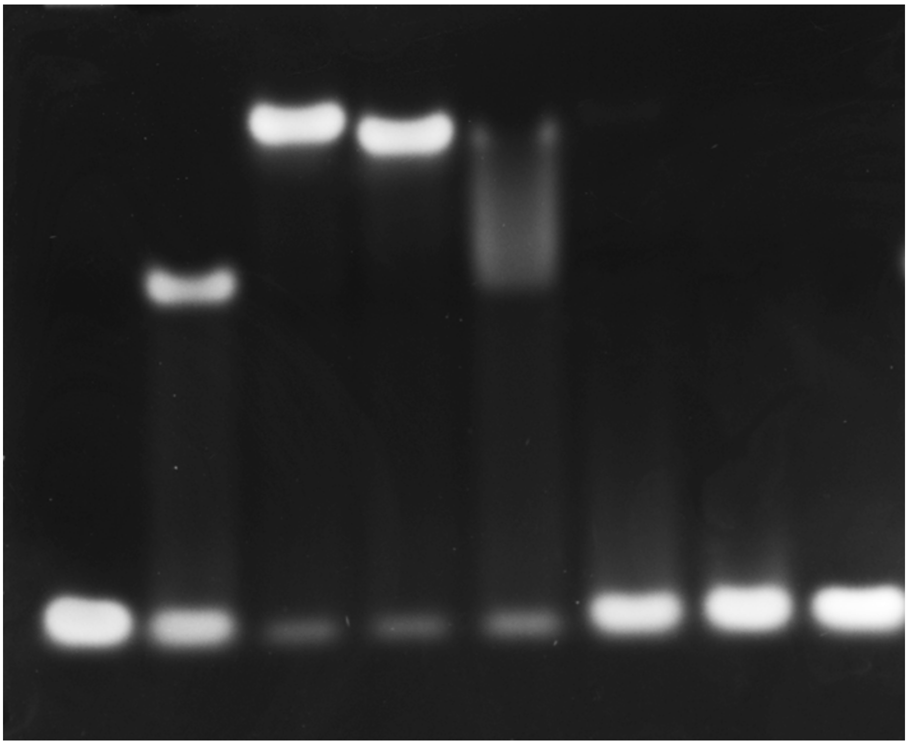

Supplementary Fig. 2a:

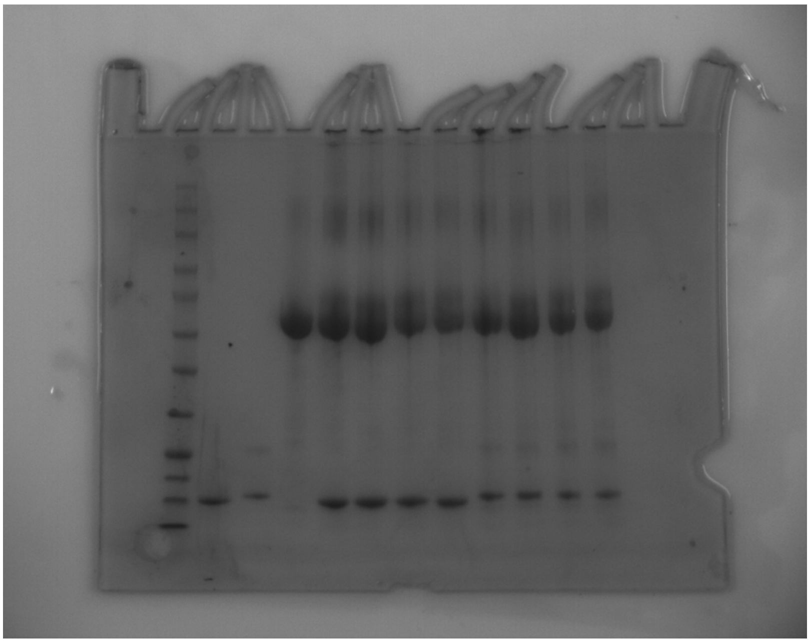

Supplementary Fig. 15a:

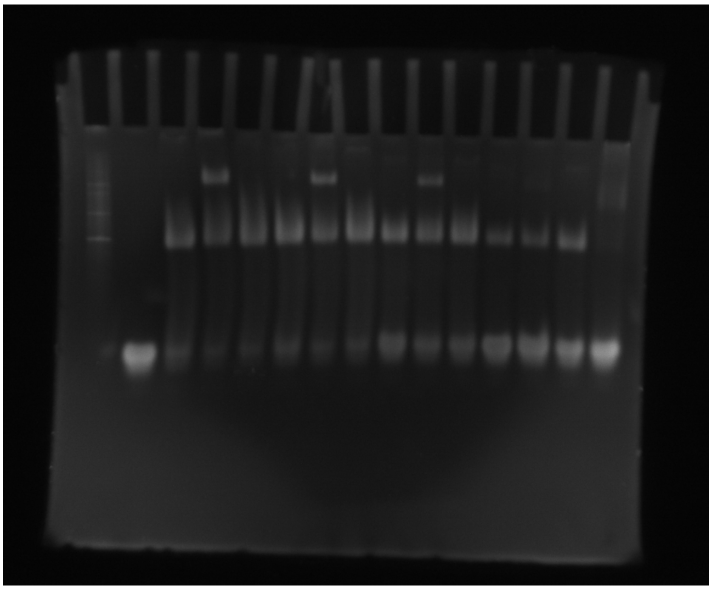

Supplement: Supplementary file 2 — Supplemental Information [file 42003_2024_6258_MOESM2_ESM.pdf]
