# Supplementary material for: Structural basis for the regulation of plant transcription factor WRKY33 by the VQ protein SIB1
Source: Commun Biol. 2024 May 11;7:561. doi: 10.1038/s42003-024-06258-7 (PMC11088704; doi:10.1038/s42003-024-06258-7)
Supplement: Supplementary file 3 — Description of Additional Supplementary Files [file 42003_2024_6258_MOESM3_ESM.pdf]

Description of Additional Supplementary Files

File name: Supplementary Data 1
Description: The source behind the Graphs in the paper.
